# Screening bisphenols in complex samples via a planar *Arxula adeninivorans* bioluminescence bioassay

**DOI:** 10.1007/s00216-023-04820-6

**Published:** 2023-07-17

**Authors:** Max Jaber, Martin Jähne, Michaela Oberle, Gertrud E. Morlock

**Affiliations:** 1 Institute of Nutritional Science, Chair of Food Science, and TransMIT Center for Effect-Directed Analysis, Justus Liebig University Giessen, Heinrich-Buff-Ring 26-32, 35392 Giessen, Germany; 2 QuoData GmbH, Prellerstrasse 14, 01309 Dresden, Germany; 3 Merck KGaA, Frankfurter Str. 250, 64293 Darmstadt, Germany

**Keywords:** High-performance thin-layer chromatography, HPTLC, Planar yeast estrogen screen assay, Thermal paper, Botanical, Can migrate, Bisphenol A, BPA

## Abstract

The *Arxula* yeast bisphenol screen (A-YBS) utilizes the bioluminescent *Arxula adeninivorans* yeast–based reporter cells for tailored analysis of bisphenols, one of the major endocrine-disrupting compound groups. For the first time, this bioreporter has been applied on the high-performance thin-layer chromatography (HPTLC) adsorbent surface to develop a respective planar bioluminescence bioassay (pA-YBS). The goal was to combine the advantages of HPTLC with a more selective bioassay detection for bisphenols. The performance of this pA-YBS bioluminescence bioassay was demonstrated by calculating the half-maximal effective concentration (EC_50_) of bisphenols compared to references. The EC_50_ ranged from 267 pg/band for bisphenol Z and 322 pg/band for bisphenol A (BPA) to > 1 ng/band for other bisphenols (BPC, BPE, BPF, and BPS) and references (17β-estradiol and 17α-ethinylestradiol). The EC_50_ value of BPA was three times more sensitive in signal detection than that of 17β-estradiol. The visual or videodensitometric limit of detection of BPA was about 200 pg/zone. The higher signal intensity and sensitivity for BPA confirmed the tailored bioassay selectivity compared to the existing estrogen screen bioassay. It worked on different types of HPTLC silica gel plates. This HPTLC–UV/Vis/FLD–pA-YBS bioluminescence bioassay method was used to analyze complex mixtures such as six tin can migrates, five thermal papers, and eleven botanicals. The detected estrogenic compound zones in the tin can migrates were successfully verified via the duplex planar yeast antagonist estrogen screen (pYAES) bioassay. The two bisphenols A and S were identified in one out of five thermal papers and confirmed with high-resolution mass spectrometry. No bisphenols were detected in the botanicals investigated via the pA-YBS bioluminescence bioassay. However, the botanicals proved to contain phytoestrogens as detected via the pYAES bioassay, which confirmed the tailored bioassay selectivity. This HPTLC–UV/Vis/FLD–pA-YBS bioluminescence bioassay is suited for cost-efficient analysis of BPA in complex samples, with no need for sterile conditions due to the fast workflow.

**Supplementary information:**

The online version contains supplementary material available at 10.1007/s00216-023-04820-6.

## Introduction

Bisphenol A (BPA) is a widespread industrial endocrine-disrupting compound (EDC) used extensively to manufacture polycarbonate plastics and epoxy resins, with an estimated global market size of 7.3 million tons by the end of 2023 [1]. Due to its widespread applications in food or beverage packing, coatings of food cans, toys, and thermal papers, BPA is detectable in various environmental samples such as wastewater, river water, groundwater, and even marine water [2]. It is capable of binding to different types of receptors, including estrogen and androgen receptors. Due to its endocrine activity even at low doses (picogram levels), as well as oxidative and mutagenic potential in animals and possibly also in humans, it can have multiple toxic effects [2–4]. In 2023, the European Food Safety Authority reestablished a tolerable daily intake of 0.2 ng/kg of body weight per day by decreasing the previous temporary level 20,000-fold. This corresponds to a maximum daily ingestion of 12 ng of BPA for a 60-kg person, which consumers with both average and high exposure to dietary BPA exceed [5]. Therefore, the European Commission would need to adjust the maximum permitted migration levels of BPA from food contact materials into food products from the current 0.05 mg/kg of food [6]. In 2020, the application of BPA in thermal paper had already been reduced to a maximum of 0.02%, which worked like a ban since its chemical properties are no longer rationally useable at such a low concentration [7]. However, bisphenol S (BPS) is increasingly replacing BPA due to it having similar chemical properties [8, 9].

BPA is analyzed with separation methods such as gas chromatography (limit of detection (LOD) 0.13 µg/L [10]) and high-performance liquid chromatography (HPLC, LOD 150 µg/L [11]) and is usually coupled with mass spectrometry (MS), especially high-resolution mass spectrometry (HRMS). Most physicochemical methods can detect and characterize target molecules, but they provide little, if any, information on the biological effect. Instead, in vitro and in vivo bioassays [12] provide information on biologically active compounds; however, due to ethical concerns, in vivo bioassays have largely diminished with the increased use of reporter cell lines. Since in vitro bioassays provide only a sum parameter value for a complex sample and are limited in how well they detect, a safety assessment is challenging because thousands of compounds (unknown unknowns) can be present in a complex sample, and opposing signal responses can cancel out important effects. Therefore, chromatographic separation is essential for multi-component mixtures to avoid interference with biological responses. Planar chromatographic separation coupled with effect-directed analysis (EDA) helps separate them from matrix interference and prioritizes compounds with biological effects from thousands of substances in a complex sample [13]. Compared to other instrumental techniques, sample preparation can be reduced to a minimum, which includes the largest possible quantity of sample components in the screening and is comparatively less time-consuming and less labor-intensive [11]. This also places high requirements on biological effect detection. It must have a high tolerance range for various environmental influences, and at the same time, it must be able to sensitively detect traces of EDCs. The *Arxula adeninivorans* yeast strain tolerates several environmental influences and can exploit a broad range of carbon and/or nitrogen sources, which is unique and beneficial for its application [14]. It has already been developed to detect estrogenic activity in liquid samples, in particular, salty samples with up to 5% sodium chloride as found in seawater and many polluted industrial wastes [14–16]. Usually, any compound binding to the receptor can activate it, and therefore the sum of all activity is measured, which is often used in screening to provide an overview. For in vitro bioassays, opposing signals unfortunately can lead to misinterpretation (as the separation is missing), and individual active chemical compounds cannot be assigned [17]. Only a tedious bioassay-guided fractionation combined with HRMS can reveal which chemical is involved. The large number of receptor-activating compounds (unknown unknowns) that we do not know about pose a further challenge.

The planar yeast estrogen screen (pYES) both chromatographically separates and detects individual estrogen-effective compounds using *Saccharomyces cerevisiae* modified to contain hERα [18–20]. However, despite the strong endocrine activity of BPA, the pYES receptor cannot detect low concentrations of BPA, which is about five orders of magnitude less sensitively detected than 17β-estradiol (E2) [21]. This is due to BPA being 3000 times less affine to hERα than E2 [19]. To overcome the low affinity of BPA towards hERα, whole-cell bioreporters can be genetically modified via directed evolution to generate a mutant receptor with the desired ligand-binding properties [22], e.g., by utilizing the bisphenol A-targeted receptor (BPA-R) [21]. A screen with positive response for BPA induction and negative response for E2 induction was targeted. The resulting target receptor in *S. cerevisiae* was fourfold more sensitive to BPA and 166,000-fold less sensitive to native ligands such as phytoestrogens [23]. This leads to a chemical class-selective yeast estrogen screen assay, which could prove useful for high-throughput screening of food, supplements, botanicals, and environmental samples.

In this study, for the first time, a new planar bioluminescent *A. adeninivorans* yeast bisphenol screen (pA-YBS) bioassay has been developed and combined with high-performance thin-layer chromatography (HPTLC) to directly run on thin-layer chromatography plates and sensitively detect individual bisphenols via the more selective (tailored) bisphenol target receptor. The pA-YBS transactivation assay uses recombinant *A. adeninivorans* yeast cells as the biosensor responsive to bisphenol. A receptor gene cassette carrying the gene for BPA-R and a reporter gene cassette carrying the modified gene for firefly luciferase from *Photinus pyralis* have been stably integrated into the yeast genome according to the procedure described by Hahn et al. [16]. After passing the yeast cell wall, bisphenols bind to single receptor molecules, inducing the dimerization of receptor molecules and initiating the transactivation mechanism in the nucleus. The biological response is detected by measuring bioluminescence that results from ligand-dependent production of firefly luciferase, which converts luciferin to oxyluciferin in the yeast cells. The performance of this HPTLC–UV/Vis/FLD–pA-YBS bioluminescence bioassay method was demonstrated by screening EDCs in six tin can migrates, five thermal papers, and eleven botanicals.

## Materials and methods

### Chemicals and materials

Double-distilled water was prepared using a Heraeus Destamat Bi-18E (Thermo Fisher Scientific, Dreieich, Germany). Bisphenol A (BPA, > 97%) and 4-*n*-nonylphenol (NP, 98%) were from Alfa Aesar, Karlsruhe, Germany. 17β-estradiol (E2, 98.5%) and 17α-ethinylestradiol (EE2, 99%) were ordered from Dr. Ehrenstorfer, Augsburg, Germany. Sigma-Aldrich in Steinheim, Germany, delivered bisphenol C (BPC, > 99%), bisphenol E (BPE, > 98%), bisphenol F (BPF, > 98%), and bisphenol G (BPG, > 98%). Bisphenol S (BPS, > 98%) and bisphenol Z (BPZ, > 99%) were purchased from Fluka Sigma-Aldrich, Steinheim, Germany. The lyophilized cells of the yeast strain *Arxula adeninivorans* G1212/YIEC-69-TEFmhERα-PHO5-GAA2ERE107-luc-PHO5 and chemicals to prepare reactivation medium and culture medium were purchased from new_diagnostics, Berlin, Germany. All solvents (chromatography grade), trisodium citrate dihydrate (> 99%), citric acid (> 99.5%), and sodium hydroxide pellets (> 99%) were from Carl Roth, Karlsruhe, Germany. D-Luciferin sodium (> 99%, 10 mM) and adenosine triphosphate (ATP, > 98%, 100 mM) were purchased from GoldBio, St Louis, MO, USA. Merck in Darmstadt, Germany, supplied TLC aluminum foil silica gel 60, HPTLC plate silica gel 60 with F_254_, and without. Differently coated non-commercial R&D tin cans (Ceritec SRL, Metlac Group, Italy) were obtained in collaboration with Nestlé Research, Vers-chez-les-Blanc, Switzerland. Thermal papers were collected from various local retail chains and sales companies in Germany. Dried botanical powders were obtained from Martin Bauer Group, Vestenbergsgreuth, Germany.

### Standard, buffer, and substrate solutions

Methanolic stock solutions of 1 µg/µL were prepared for all standard compounds (S0) and diluted up to 1:10,000,000 to obtain 100 ng/µL (S1), 10 ng/µL (S2), 1 ng/µL (S3), 100 pg/µL (S4), 1 pg/µL (S5), and 0.1 pg/µL (S6), whereas the S5 and S6 dilutions were only needed for BPA, BPZ, and E2 solutions. To prepare the 0.1 M sodium citrate buffer, 138 mg of sodium citrate dihydrate and 872 mg of citric acid were weighed out and dissolved in a Falcon tube, containing double-distilled water. The pH was then adjusted to 3 with solid sodium hydroxide. The 2.3 mL luciferin substrate solution was freshly prepared before each use. Therefore, frozen luciferin and ATP solutions were thawed at room temperature, and sodium citrate buffer (1.61 mL), luciferin (0.23 mL), and ATP (0.46 mL) were mixed to a final concentration of 1 mM luciferin and 20 mM ATP.

### Sample preparation

Three different types of samples were prepared (Table S1). Each tin can was filled with 300 mL of food simulant solvent (ethanol 95%), closed with 50-µm-thick aluminum foil (Korff, Oberbipp, Switzerland), and placed in an incubator at 60 °C for 10 days to produce the tin can migrate, which was then concentrated 30-fold (30 ×) by evaporating the solvent from an aliquot (30 mL) under nitrogen atmosphere and resolving the residue in 1 mL of food simulant solvent (ethanol 95%) [24, 25]. Each thermal paper was cut into small pieces *circa* 5 mm × 5 mm, weighed (0.5 − 1 g), extracted with methanol (4 − 6 mL) on a vortex for 2 h (0.13 − 0.20 g/mL), and centrifuged at 3000 × *g* for 5 min [26]. Each botanical extract (0.5 g each) was suspended in 5 mL methanol (0.1 g/mL), ultrasonicated for 30 min, and centrifuged at 3000 × *g* for 15 min [20]. Each sample solution was transferred into an autosampler vial.

### Preparing the cell suspension

The lyophilized pellet of *A. adeninivorans*–based BPA-R reporter strain cells (OD_620_ of 10) was suspended in a 1.6-mL reactivation medium, homogenized, and subsequently centrifuged (3000 × *g*, 30 s). The supernatant was carefully decanted and discarded. After repeating this washing procedure twice more with 1 mL of reactivation medium each time, the yeast cells were resuspended in 1 mL of culture medium. An aliquot (0.4 mL) was added to the culture medium (10 mL) in a 50-mL Erlenmeyer glass flask with a baffle and incubated at 30 °C by shaking at 100 rpm for 18–24 h. In the first experiments, the cell culture was split (1:10) to have the cells present again in the exponential growth phase for the following day.

### Band pattern pre-test

Solvent blank and 5 ng (0.5 µL S2), 50 ng (0.5 µL S1), and 250 ng BPA (2.5 µL S1) were manually applied onto the TLC foil silica gel 60 as spots with a 1-µL capillary. The cell suspension was applied in three different modes, i.e*.*, by (I) manually dipping the plate stripe with tweezers into the immersion chamber (10 cm × 10 cm, biostep, Burkhardtsdorf, Germany) filled with 10 mL of cell suspension for 3 s, (II) spotting 10 µL of cell suspension with a 20-µL pipette onto each applied BPA spot, and (III) piezoelectrically spraying the cell suspension onto the plate (red nozzle, level 6, Derivatizer, CAMAG). On-surface incubation took place in a closed water bath at 37 °C for 1, 2, and 3 h. For this, the plate back was fixed with magnets to a perforated metal plate where the adsorbent layer faced the rising water vapor 3 cm from the water surface (Fig. S1). After plate drying in a cold stream of air for 1 min, the luciferin substrate solution was applied in the same mode and incubated again on the surface as described (37 °C, 15 min), and bioluminescence was recorded for 1 min (BioLuminizer, CAMAG).

BPA from 100–800 pg per 6-mm band (1–8 µL S4) was applied three times onto the HPTLC plate silica gel 60 F_254_ as a band pattern, i.e*.*, one band above the other at increasing volumes/amounts (Automatic TLC Sampler ATS4 with Freemode option, CAMAG) and dried (no plate development). The cell suspension, incubation, and substrate application were piezoelectrically sprayed as described.

### Determining the half-maximal effective concentration (EC_50_)

The respective reference solutions were applied onto the HPTLC plate silica gel 60 as 6-mm band patterns at the indicated amount range. After the bioassay, the bioluminescence image (10-min exposure time, bioluminescence depicted as greyscale image) was used to extract the videodensitograms of the individual tracks. For this purpose, the image file (.bmp) was loaded into VideoScan software (CAMAG) and evaluated (integrated) to obtain the peak area for each zone of interest. Using the Hill function, three-parameter calibration curves were determined, and the EC_50_ values were calculated with the freeware EC_50_ Calculator (AAT Bioquest, Sunnyvale, CA, USA).

### HPTLC–UV/Vis/FLD–pA-YBS bioluminescence bioassay method

The tin can migrate 30 × concentrates, thermal paper extracts (10 µL each), and botanical extracts (7 µL each) were applied as bands (tin can migrate 30 × concentrates 8 mm; botanical extracts 5 mm) or as an area (thermal paper extracts 5 mm × 3 mm) as indicated onto the HPTLC plate silica gel 60 with or without F_254_ along with the respective solvent blank (B) and reference standard (ATS4, CAMAG). Thermal paper extracts were applied in duplicate, and every second one was oversprayed with 10 µL BPA solution (S3; 10 ng/band; marked *). After drying the application zones in a cold stream of air for 1 min, the plate was developed in the Twin-Trough Chamber (10 cm × 10 cm, or 20 cm × 10 cm, CAMAG). Toluene – ethyl acetate 6:1, *V/V*, was used to separate tin can migrate 30 × concentrates, whereas ethyl acetate – toluene – methanol – water 16:4:3:2, *V/V/V/V*, separated the botanical extracts, both up to a migration distance of 70 mm. The thermal paper extracts applied as areas were first focused with ethyl acetate – cyclohexane 1:1, *V/V*, and then developed with *n*-hexane – ethanol – ethyl acetate 4:0.3:0.3, *V/V/V*, up to a migration distance of 60 mm. After plate drying in a cold stream of air for 4 min, the chromatogram was documented at white light illumination (Vis), UV 254 nm, and FLD 366 nm (TLC Visualizer, CAMAG).

For biological detection, the yeast cell suspension was piezoelectrically sprayed (3.1 mL, red nozzle, level 6, Derivatizer, CAMAG) onto the chromatogram. Incubation took place in a closed water bath at 37 °C for 2 h. After plate drying in a cold stream of air for 5 min, the plate was piezoelectrically sprayed with the substrate solution (2.3 mL, yellow nozzle, level 2, Derivatizer, CAMAG) and incubated (37 °C, 15 min). The enzyme − substrate reaction was terminated by plate drying, and the emitted bioluminescence was recorded instantly using exposure times of 1 min and then 10 min (BioLuminizer, CAMAG). All instrumentation was operated via visionCATS software (version 3.2, CAMAG).

### Recording the HPTLC–HESI-HRMS spectra

The thermal paper extract V was applied in duplicate as a 3 mm × 5 mm area at five increasing volumes (10, 20, 30, 40, and 50 µL) on an HPTLC plate. After development and plate cut, one plate stripe was detected via the pA-YBS bioluminescence bioassay, and the other plate stripe was used to record HRMS spectra. Zones of interest were eluted (methanol, flow rate 0.2 mL/min, Dionex Ultimate 3000 UHPLC system, Thermo Fisher Scientific) using a TLC-MS Interface 2 equipped with an oval elution head 4 mm × 2 mm [27] (CAMAG) for an elution time of 60 s and transferred to the Q Exactive Plus Hybrid Quadrupole Orbitrap Mass Spectrometer (Thermo Fisher Scientific) equipped with an Ion Max heated electrospray ionization (HESI)-II probe with spray voltage 3.5 kV, aux gas 10 arbitrary units (AU), sheath gas 20 AU, probe heater temperature 200 °C, capillary temperature 270 °C, and S-lens RF level 50 AU. A filter frit (Upchurch Scientific A-356 and PEEK-Frit Blue UPA-703, Techlab, Erkerode, Germany) was installed in the interface outlet line to the mass spectrometer to protect the HESI source from particles. Full scan HRMS spectra (*m*/*z* 100–1000) were acquired in the positive and negative ionization modes (resolution of 280,000 at *m*/*z* 200 for full width at half maximum). The system used Xcalibur 4.2.47 with Foundation 3.1.261.0 and SII for Xcalibur 1.5.0.10747 software (Thermo Fisher Scientific). External mass calibration was performed daily (Pierce LTQ Velos ESI positive/negative ion calibration solution, Thermo Scientific). System/plate blank spectra were recorded in between the analyte spectra recordings for subtraction.

## Results and discussion

HPTLC was the preferred method since complex samples needed minimal preparation (high matrix tolerance), could be enriched during spray-on application (solvent evaporation), and were separated from the matrix (important when differentiating opposing biological signal responses as discussed in the examples). Using the *A. adeninivorans*–based BPA-R reporter strain, any bisphenol or estrogenic compound present should have been detected as a bioluminescent response signal since the applied luciferin substrate released and detected luciferase upon receptor binding. Luciferase acted on the luciferin substrate to produce oxyluciferin in an electrically excited state, which released a photon of light as it returned to the ground state and was bioluminescent at 550–570 nm at low pH values.

### Study of three cell application techniques and different plates

**Fig. 1:**
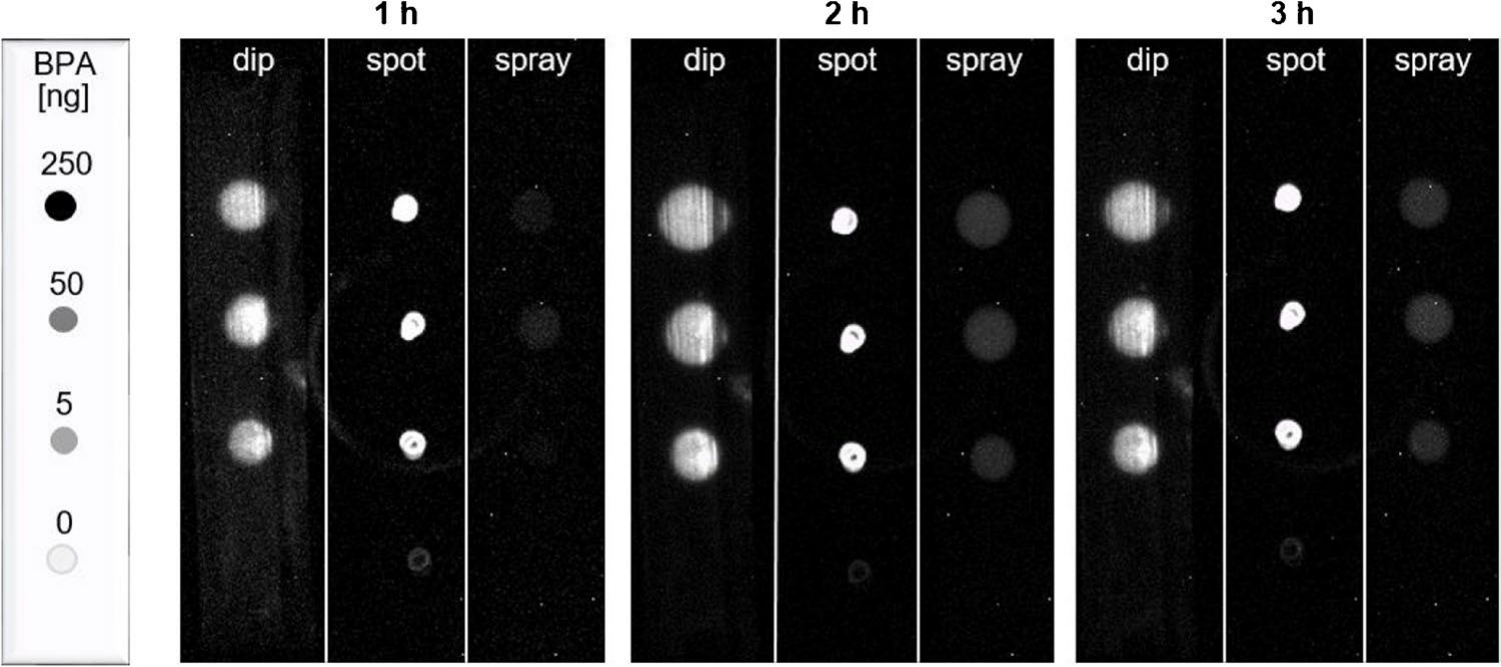
Study of three cell application techniques for the pA-YBS bioluminescence bioassay: manual dipping, spotting, and automated spraying of the cells on the TLC foil silica gel 60 were compared regarding the bioluminescence signal of BPA (0–250 ng/zone) measured after 1 h, 2 h, and 3 h incubation time (bioluminescence depicted as a greyscale image); upon receptor binding of BPA in the *A. adeninivorans*–based BPA-R reporter strain, the luciferase was released and was subsequently detected once the luciferin substrate produced bioluminescent oxyluciferin

One objective of this planar bioassay development was to study the feasibility of running the assay in simply equipped laboratories. Therefore, three different cell application modes (manual dipping, spotting, and automated spraying) were compared on this yet untested bioluminescent *A. adeninivorans–*based BPA-R reporter strain. Manually spotting the cell suspension on top of the applied sample bands is the simplest method; however, it lacks precision and is user dependent, whereas both manual and automated spraying of the cell suspension [28, 29] or immersion into it [15, 30] are possible. Additionally, on-surface incubation in a simple water bath instead of a more expensive incubator was investigated for three different durations (1, 2, and 3 h). As a result, the applied BPA spot pattern (0 − 250 ng/band) showed bioluminescence signal responses for all three cell application techniques, albeit with very different response intensities (Fig. 1). For the applied BPA amounts, both dipped and spotted cells showed a bioluminescence signal for BPA after merely 1-h of on-surface incubation, whereas the sprayed cells showed only a very weak bioluminescence signal that slightly increased with longer incubation times. This difference was due to the lower cell concentration or coverage per area than in the immersion. The entire plate strip was covered with cells via dipping and spraying, and the detected bioluminescent BPA zone diameter increased with the amount. However, for the plate stripe with the spotted yeast cells, an increase in the bioluminescence signal with the increasing BPA amount was hardly detectable, which was due to the capillary diameter since the cells spotted on the BPA maximum did not diffuse on the adsorbent and the signal was already saturated for the high BPA amounts. Comparing all results, the cell spotting technique was rejected since the cells could not be applied uniformly over the whole BPA zone. The immersion technique was suitable but was also rejected since it required a high cell suspension volume for dipping the whole plate (high consumable costs) and can cause zone blurring or tailing. Though weakest in the response, the spraying technique was nonetheless selected in combination with a 2-h incubation time. In contrast, comparable bioassays such as the pYES or the duplex planar yeast antagonist estrogen screen (pYAES) need a 3-h incubation [8, 9, 20, 30]. Note that signal intensity is always a compromise between time and zone diffusion.

Studying different types of HPTLC silica gel plates, a plate influence was minor comparing plates without fluorescence indicator, with fluorescence indicator F_254_ and with acid-stable fluorescence indicator F_254_ s (Fig. S2). Since the bioluminescent bioassay worked on the different silica gel plates, its flexibility regarding the selection of the silica gel plate was given. However, signal intensity was comparatively lower on HPTLC silica gel AMD plates which are reduced in layer thickness and more acidic compared to regular silica gel plates, which explained the lower sensitivity.

### Study of the dose–response and determination of the EC_50_ of BPA

**Fig. 2:**
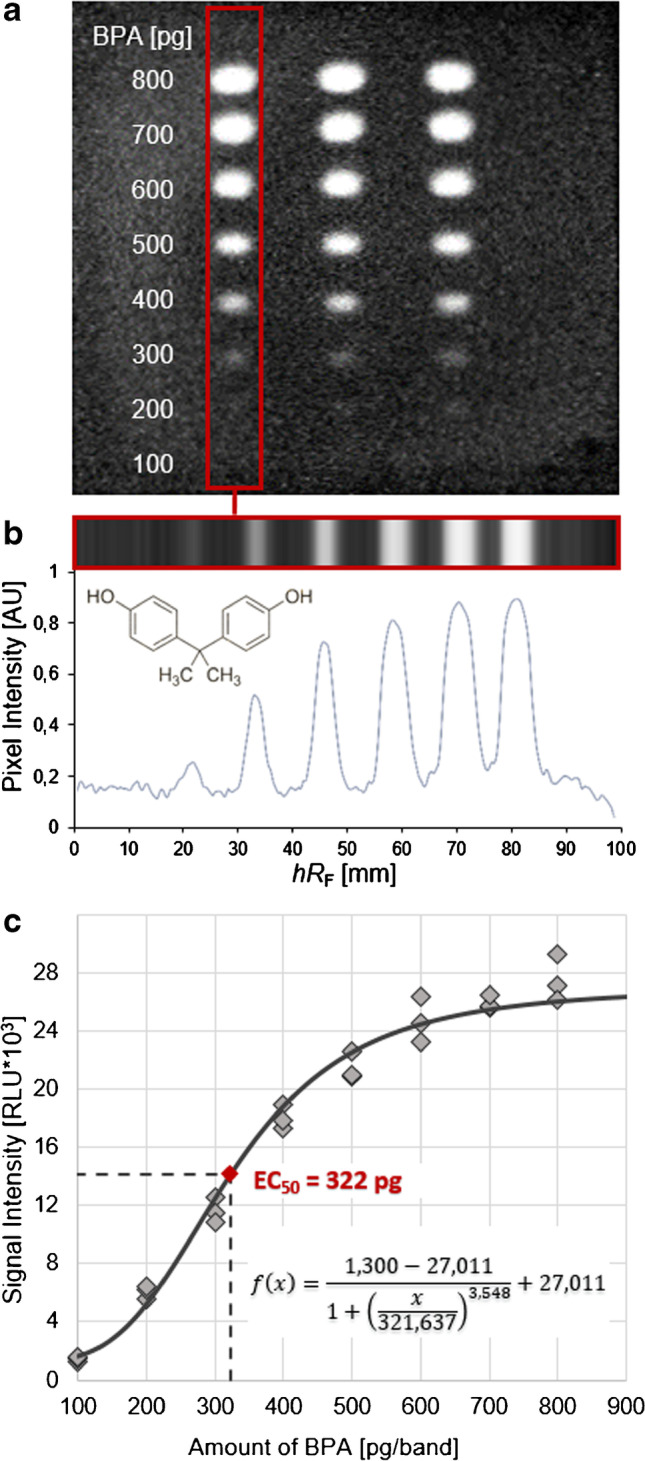
Study of dose–response and determination of the EC_50_ of BPA (100–800 pg/band, *n* = 3) on the HPTLC plate silica gel 60 F_254_ using the pA-YBS bioassay: **a** bioluminescence image for a 10 min exposure (bioluminescence depicted as a greyscale image), **b** track used to generate the densitogram, and **c** calculated dose–response curve of BPA showing the EC_50_ of BPA at 322 pg/band

**Fig. 3:**
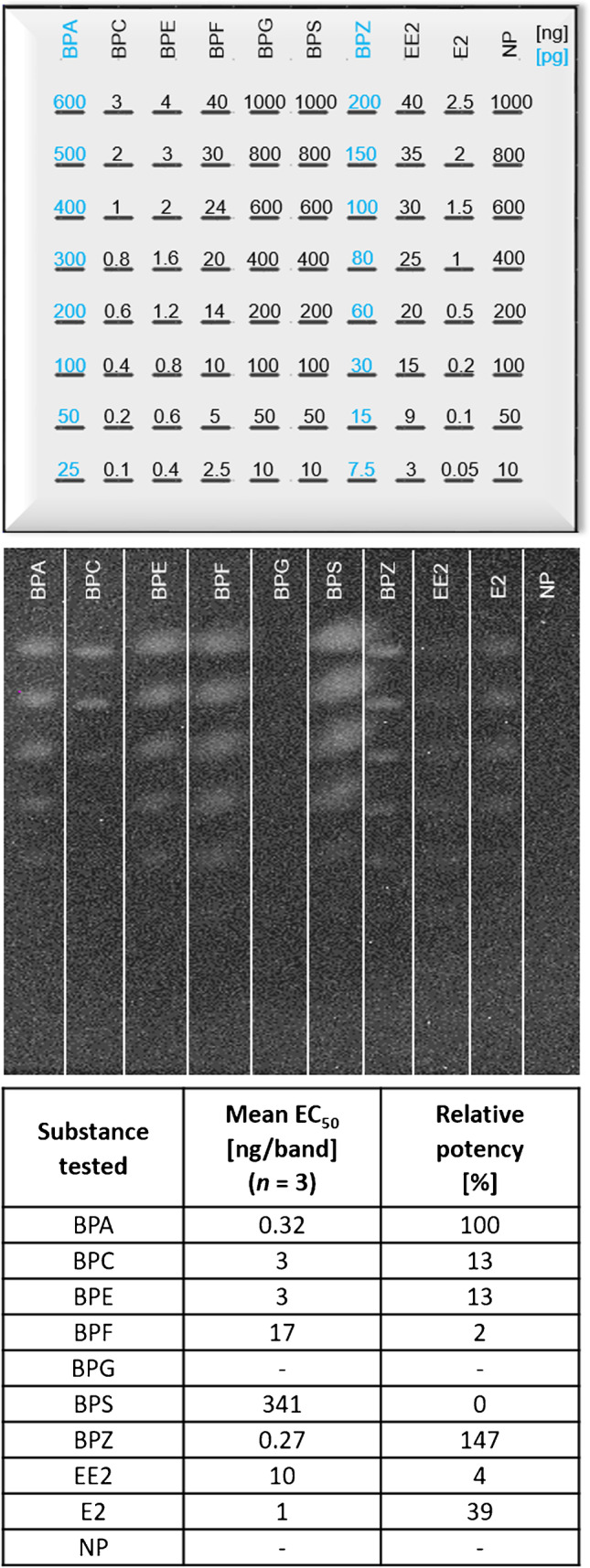
Selectivity, EC_50_ of further compounds, and relative potency towards BPA: application scheme for different application amounts (pg/band or ng/band, depending on sensitivity) of six bisphenols (BPA, BPC, BPE, BPF, BPG, BPS, and BPZ), two estrogens (EE2, and E2), and NP on the HPTLC plate silica gel 60 and corresponding bioluminescence image for a 10-min exposure time (bioluminescence depicted as greyscale image) used to calculate the EC_50_ values and relative potency towards BPA

The EC_50_ value was determined for BPA applied at seven different amounts on the HPTLC plate (Fig. 2 and Fig. S2). After the cell suspension was sprayed, incubated for 2 h, dried, sprayed with the luciferin substrate solution, and incubated for 15 min, the bioluminescent bioautogram was recorded (Fig. 2a), from which the videodensitograms were extracted (Fig. 2b). The visual LOD of about 200 pg/zone was comparable to the densitometric LOD. The calibration curve obtained from the Hill function was used to calculate the EC_50_ value of 322 pg/band for BPA (Fig. 2c), which was three times more affinitive on the receptor than E2 (1 ng/band, Fig. 3). In contrast, the previously used hERα had 3000 times less receptor affinity on BPA than E2 [19]. This improvement in the sensitivity of BPA was in agreement with other studies comparing BPA-R and hERα receptors [23].

### Selectivity, EC_50_ of additional compounds, and relative potency towards BPA

To compare the selectivity and sensitivity of the newly developed pA-YBS luminescence bioassay, six additional bisphenols (BPC, BPE, BPF, BPG, BPS, and BPZ), two estrogens (EE2, and E2), and NP were analogously tested to obtain EC_50_ values and their relative potency towards BPA (Fig. 3). The EC_50_ values at 322 pg/band for BPA and 267 pg/band for BPZ were the lowest, whereas other bisphenols were about 7–50 orders of magnitude less potent inducers. This was advantageous for assay selectivity since interfering signals from other chemicals than BPA allowed for minimalistic sample preparation. The EC_50_ order of the tested chemicals increased as follows: BPZ < BPA < E2 < BPC < BPE < EE2 < BPF < BPS << BPG/NP (Fig. 3). The respective visual LOD value can be deduced from the image of the band pattern study. Directional evolution of the hERα receptor, shown for the first time in *A. adeninivorans*, increased affinity for BPA and BPZ and decreased affinity for E2 and other bisphenols. In agreement with our results, BPS, BPF, and E2 have already been shown to be less potent inducers of BPA-R than hERα in *S. cerevisiae* [23].

### The HPTLC–UV/Vis/FLD–pA-YBS bioluminescence bioassay method

**Fig. 4:**
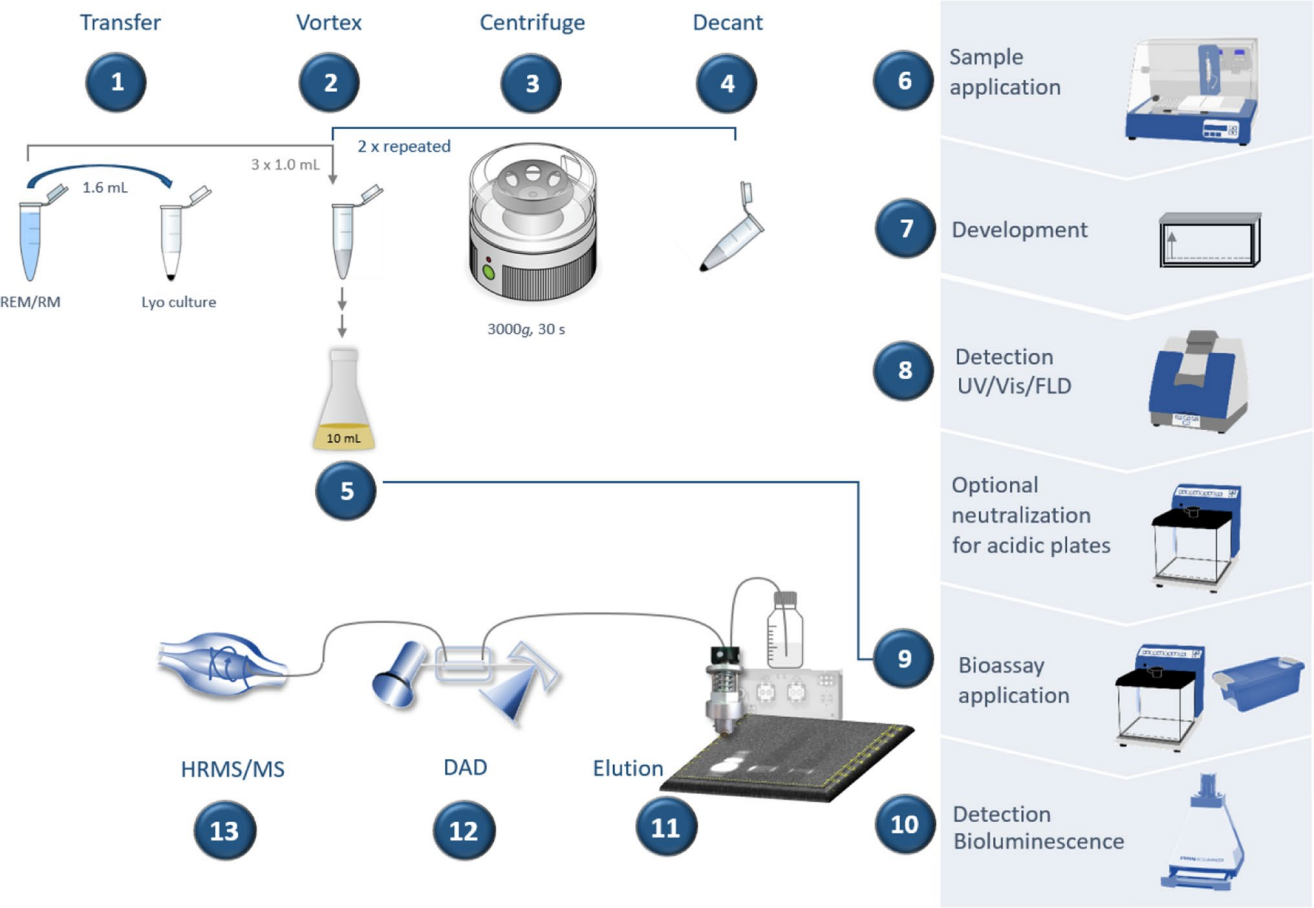
Workflow scheme of the HPTLC–UV/Vis/FLD–pA-YBS bioluminescence bioassay method: purification and reactivation of the lyophilized cells (#1–5), sample application, plate development, and detection (#6–8), followed by biological detection (#9/10) and DAD–HRMS/MS detection (#11–13); the whole analysis took 4 h (18 min per sample)

After this basic proof of principle, our planar bioassay was combined with chromatographic separation to directly detect estrogenic effects caused by bisphenols in a complex mixture. Examples analyzing six tin can migrates, five thermal papers, and eleven botanicals were given as follows. The workflow of the HPTLC–UV/Vis/FLD–pA-YBS bioluminescence bioassay was straightforward (Fig. 4). The lyophilized yeast cells were washed and incubated for 18–24 h (Fig. 4, #1–5). On the next day, samples were applied, simultaneously separated, and detected via UV/Vis/FLD (Fig. 4, #6–8). For biological detection (Fig. 4, #9/10), the yeast cell suspension was homogeneously sprayed onto the adsorbent surface and incubated for 2 h. Upon receptor binding of a bisphenol, the luciferase was released during this incubation. The luciferin substrate solution was sprayed onto the plate, followed by another incubation for 15 min. This time, the luciferase acted on the luciferin substrate to produce oxyluciferin. This enzyme − substrate reaction was terminated by drying the plate, but it could be reactivated by re-wetting the plate the very next day (Fig. S3). Its bioluminescence was recorded for 1-min and then 10-min exposure times to obtain two bioautograms. The short 1-min exposure was to see whether the bioluminescence reaction was measurable; if so, the 10-min exposure image was taken to receive the maximum light yield and therefore maximize detectability. Additionally, longer exposure times did not improve signal detection. The whole analysis took 4 h (18 min per sample). To characterize zones of interest via the respective HRMS signals in the positive/negative ionization mode, the zones were eluted and transferred to the DAD–HRMS/MS system (Fig. 4, #11–13).

### Analysis of six tin can migrates and comparison with the pYAES bioassay

**Fig. 5:**
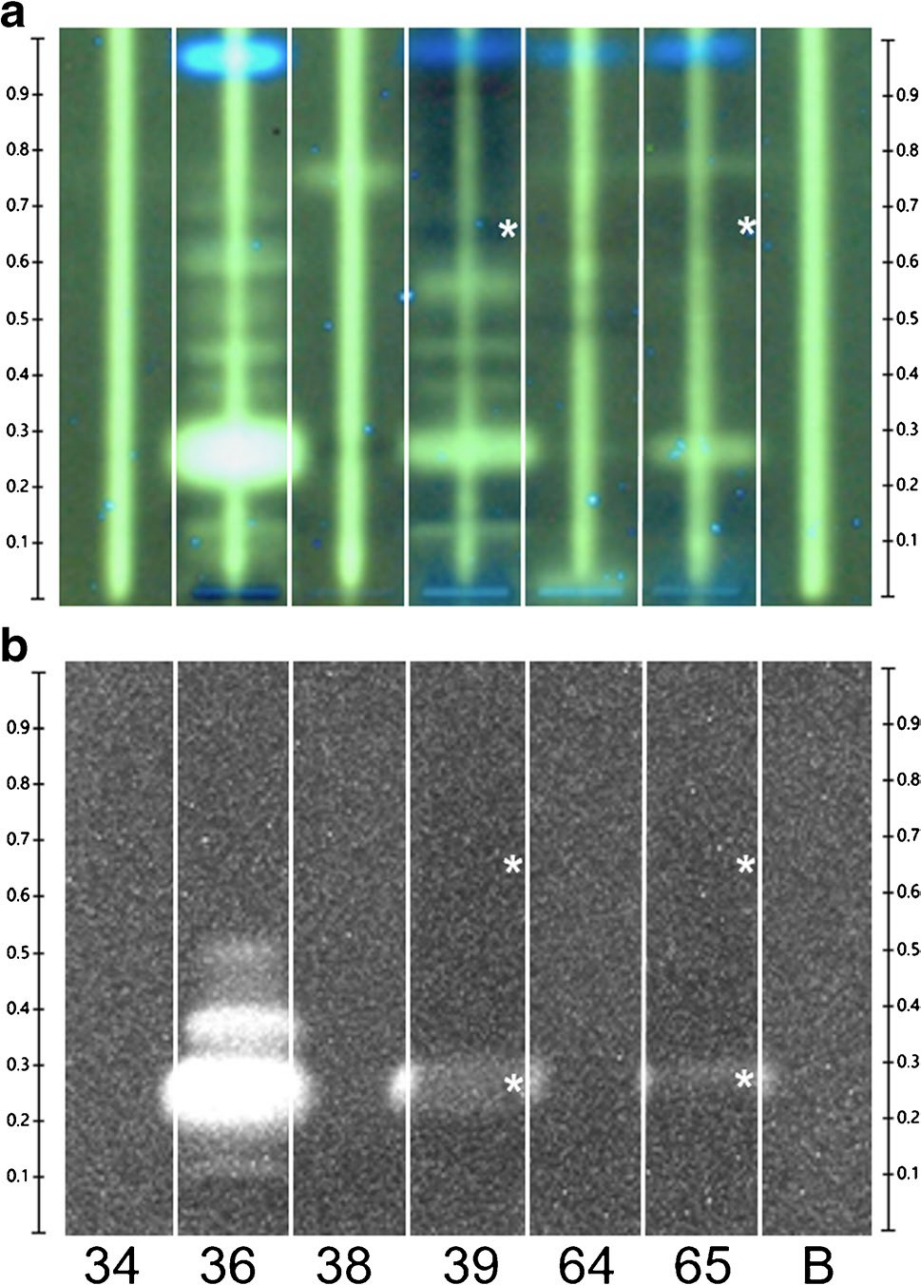
Bioautograms of the six differently coated tin can migrates (Table S1: 34, 36, 38, 39, 64, and 65; 10 µL/8 mm band) along with the food simulant blank (B) separated on the HPTLC plate silica gel 60 with toluene – ethyl acetate 6:1, *V/V*, and detected **a** at 254 nm via the pYAES bioassay with E2 agonist stripe (5.7 µL) to detect respective antagonists [25] and **b** pA-YBS bioluminescence bioassay (10 min exposure time, bioluminescence depicted as greyscale image); some compounds showed response signal reduction or quenching (marked *)

Migrates of six differently coated R&D tin cans filled with food simulant were produced, concentrated, and analyzed. Tin can migrate 30 × concentrates nos. 36, 39, and 65 showed activation of the bioluminescent *A. adeninivorans*–based BPA-R reporter strain (Fig. 5b). This fully agreed with previous results using the hERα receptor cell strain in the pYAES bioassay (Fig. 5a, [25]). In both bioassays, the intensity of the response signal at *hR*_F_ 25 was highest for the tin can migrate 30 × concentrate no. 36 and was tentatively assigned to epoxidized octadecanamide resins [25], which are used in can coatings together with bisphenols [31]. However, this zone was also present in the tin can migrate 30 × concentrates no. 39 and 65, though it was weaker and had a kind of quenching or reduced bioluminescence in the band’s middle (Fig. 5b, marked *). It was unclear whether this response reduction by other sample compounds is an antagonistic effect on the receptor (biological effect), a physicochemical reduction, or a cytotoxic effect. This sort of response reduction was also detected by reducing the fluorescence of the agonist stripe in the pYAES bioassay (Fig. 5a). Compared to this bioassay, which requires a fluorogenic substrate and must exclude any false-positive fluorescent responses, only compounds activating the *A. adeninivorans*–based BPA-R reporter showed a bioluminescence response in our pA-YBS bioluminescence bioassay, which makes it more selective. Moreover, we observed that other compounds could quench bioluminescence in the tin can migrate 30 × concentrates nos. 39 and 65 (Fig. 5b).

### Analysis of five thermal papers and zone identification via HPTLC-HESI-HRMS

**Fig. 6:**
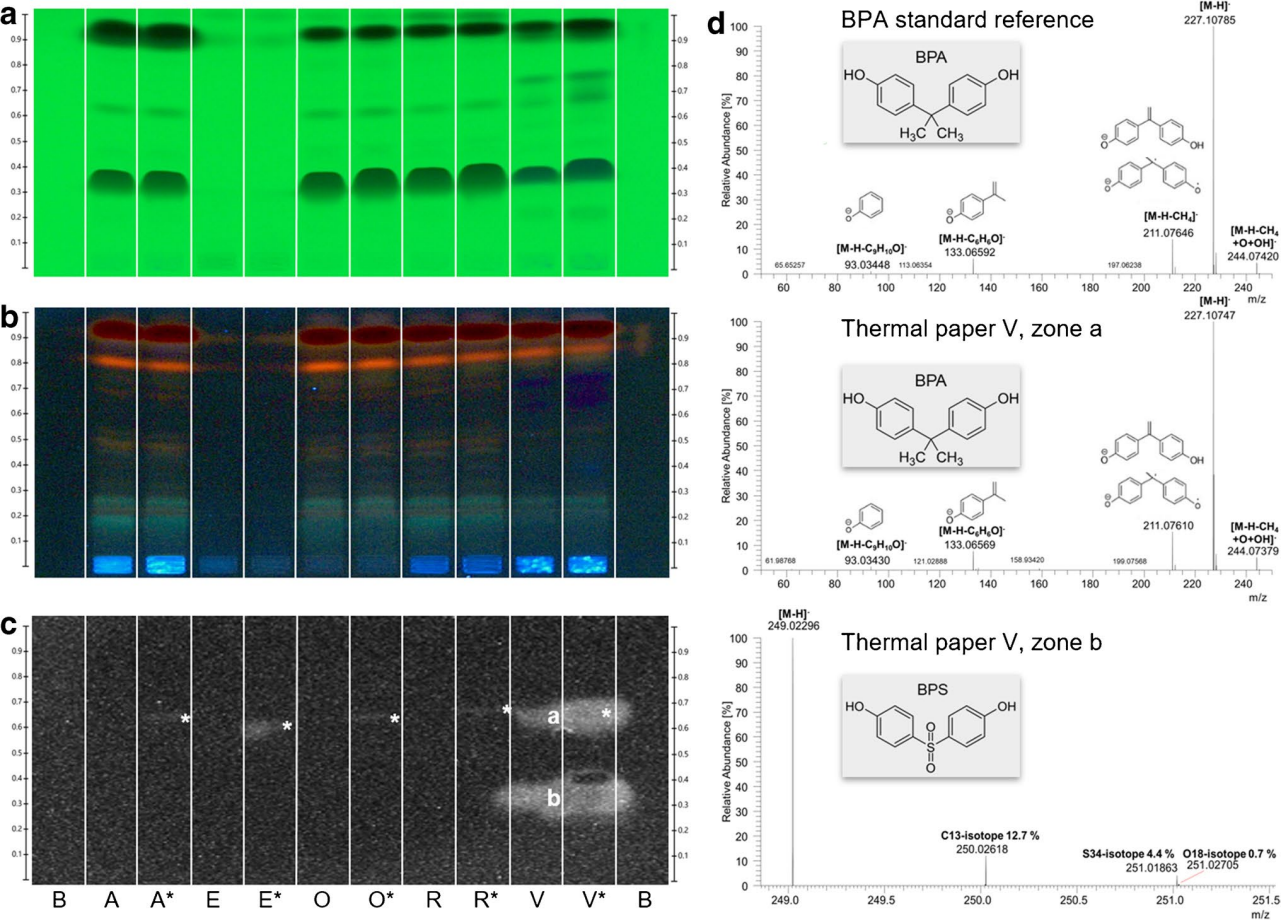
HPTLC chromatograms and bioautograms of the five different thermal paper extracts (Table S1: A, E, O, R, and V; 10 µL/5 mm × 3 mm area), where each second was oversprayed with BPA (marked *, 10 µL of S3, 10 ng/area each), along with the solvent blank (B) on the HPTLC plate silica gel 60 F_254_, focused with ethyl acetate – cyclohexane 1:1, *V/V*, separated with *n*-hexane – ethanol – ethyl acetate 4:0.3:0.3, *V/V/V*, and detected at **a** UV 254 nm, **b** FLD 366 nm, and **c** after the pA-YBS bioluminescence bioassay (10 min exposure time, bioluminescence depicted as a greyscale image); **d** HPTLC–HESI-HRMS spectra of the BPA reference compound (10 ng) and both estrogenic zones **a** (assigned to BPA) and **b** (assigned to BPS) in the thermal paper V (50 µL)

BPA was used to produce thermal paper until the EU-wide ban in 2020 [7]. Since then, manufacturers have switched production and use to alternative color developers such as BPS or BPF. Since the HPTLC–UV/Vis/FLD–pA-YBS bioluminescence bioassay method was expected to detect such xenoestrogens, thermal papers were procured at the cash register of five local shops (Table S1, labeled A, E, O, R, and V) and were investigated for bisphenols. Methanol was used for extraction. Along with the solvent blank (B), each thermal paper extract was applied twice, and every other one was oversprayed with BPA (marked *). The plate was developed [26] and detected at UV/Vis/FLD (Fig. 6a/b). After subsequent biological detection, BPA was detected and confirmed in thermal paper V as the bioluminescent compound zone **a**, which the BPA overspray in the bioautogram proved (Fig. 6c). Most matrices were separated from any detected BPA; however, bioluminescence quenching was apparent at *hR*_F_ 40 in thermal paper V as well as V* (Fig. 6a and c). Another bioluminescent compound zone (**b**) was detected at *hR*_F_ 35 in thermal paper V (Fig. 6c). Both estrogenic zones were further characterized by recording HPTLC–HESI–HRMS spectra (Fig. 6d). Zone **a** showed the deprotonated molecule at *m*/*z* 227.1075 [M-H]^−^ as the base peak and altogether the same signals and fragments as the reference BPA, whose signals were in agreement with those in the literature [32]. Although producing thermal paper that contains BPA was prohibited, residual thermal paper rolls were still allowed to be spent in January 2022. For zone **b**, the base peak was the deprotonated molecule at *m*/*z* 249.0230 [M-H]^−^, which was assigned to the molecular formula C_12_H_9_O_4_S corresponding to BPS [32]. This result was plausible as BPS has been used as an alternative color developer [8, 9] or in combination with BPA in thermal paper production [33].

### Analysis of eleven botanicals and proof of bioassay selectivity

**Fig. 7:**
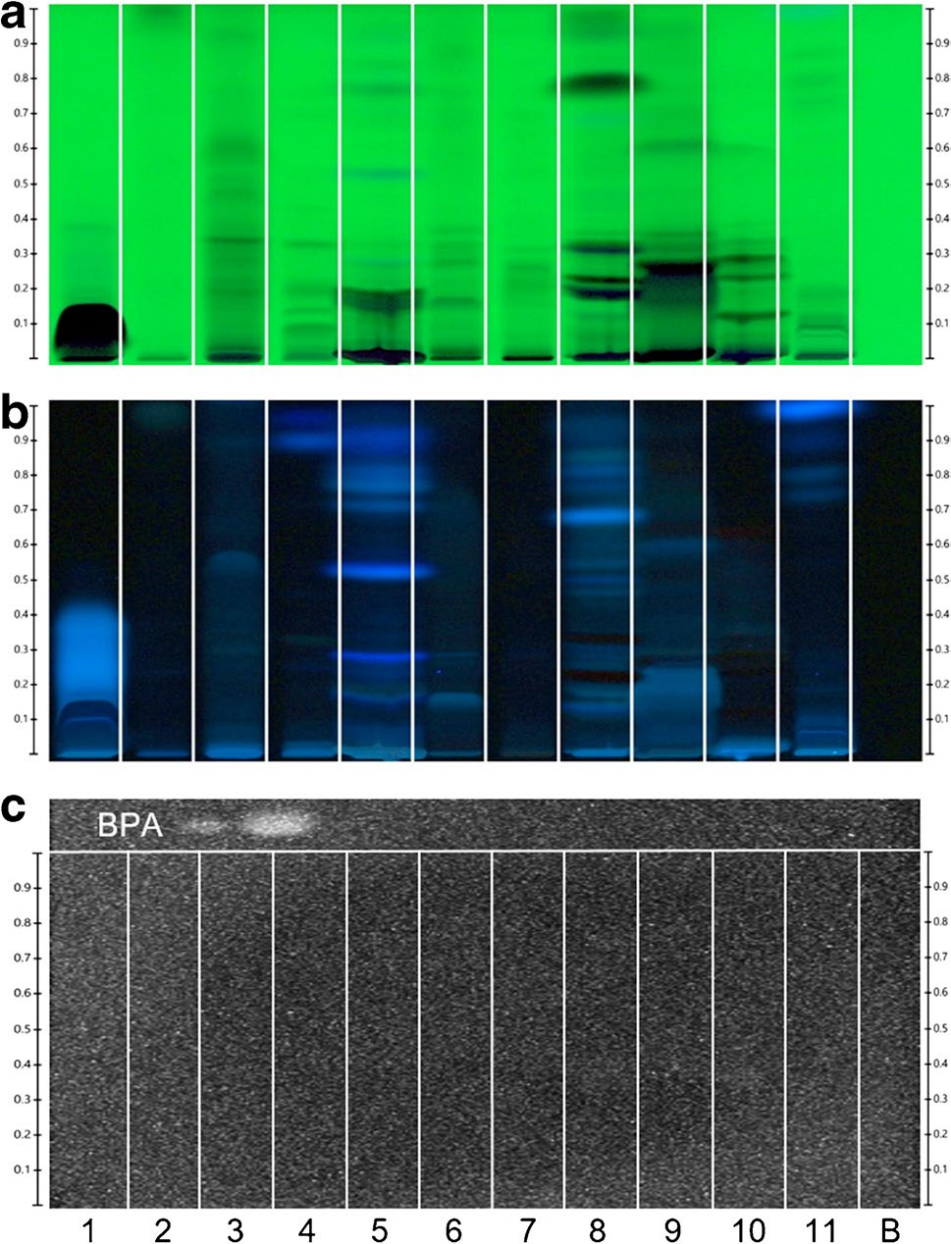
HPTLC chromatograms and bioautograms of the eleven different botanical extracts 1–11 (7 µL/5 mm band) along with the solvent blank (B) and positive control BPA (two different amounts applied on the left plate top) separated on the HPTLC plate silica gel 60 F_254_ with ethyl acetate – toluene – methanol – water 16:4:3:2, *V/V/V/V,* and detected at **a** UV 254 nm, **b **FLD 366 nm, and **c** after the pA-YBS bioluminescence bioassay (10 min exposure time, bioluminescence depicted as greyscale image)

Eleven botanicals that showed estrogenic activity using the pYES bioassay were selected [20]. These were suspended/extracted with methanol. For analysis along with the solvent blank (B), the mobile phase was slightly adjusted (the acid was substituted with methanol, Fig. 7a/b). Before the bioassay, the positive control BPA was applied at the left top plate part (2 and 5 ng/band). After the bioassay, the bioluminescence of the positive control BPA confirmed proper bioassay performance (Fig. 7c). For the eleven botanical samples, no bioluminescence signal response was detected. This result was explained by the absence of bisphenols since plant extracts primarily contain phytoestrogens, for which the BPA-R sensitivity was 166,000-fold lower for E2 than for hERα in *S. cerevisiae* cells with the luciferase reporter [21]). This confirmed our previous BPA-R affinity results for the six bisphenols, two estrogens, and NP, since the BPA-R detected E2 with a 39% relative potency to BPA. This confirmed the selectivity of this bioassay towards bisphenols which can be advantageous when screening environmental samples [34].

## Conclusions

The powerful potential of combining two disciplines (chemistry and biology) on the same surface was successfully demonstrated. The newly developed planar bioluminescent bioassay method for assessing the safety of complex mixtures was highly robust, fast, and cost-efficient. It allowed the detection of very low levels of BPA without the need for complex fractionation and tedious isolation. The proof of principle of this HPTLC–UV/Vis/FLD–pA-YBS bioluminescence bioassay method was successfully shown for three different categories of complex samples (six tin can migrates, five thermal papers, and eleven botanicals) using minimalistic sample preparation to allow as much sample integrity as possible and a fast screening. The *A. adeninivorans–*based BPA-R reporter strain was more sensitive to bisphenols, highly tolerant of environmental and workplace conditions (did not have to be sterile), and needed a shorter incubation time than the existing pYES bioassay.

## Supplementary Information

Below is the link to the electronic supplementary material.

Supplementary file1 (PDF 424 kb)
