# Supplementary material for: Screening bisphenols in complex samples via a planar Arxula adeninivorans bioluminescence bioassay
Source: Anal Bioanal Chem. 2023 Jul 17;415(21):5193–204. doi: 10.1007/s00216-023-04820-6 (PMC10404207; doi:10.1007/s00216-023-04820-6)
Supplement: Supplementary file 1 — Supplementary file1 (PDF 424 kb) [file 216_2023_4820_MOESM1_ESM.pdf]

Supplementary Material

Screening bisphenols in complex samples via a planar *Arxula adeninivorans* bioluminescence bioassay

Max Jaber[1,#], Martin Jähne[2], Michaela Oberle[3], Gertrud E. Morlock[1,#,*]

[1]Institute of Nutritional Science, Chair of Food Science, and TransMIT Center for Effect-Directed Analysis, Justus Liebig University Giessen, Heinrich-Buff-Ring 26-32, 35392 Giessen, Germany

[2]QuoData GmbH, Prellerstrasse 14, 01309 Dresden, Germany

[3]Merck KGaA, Frankfurter Str. 250, 64293 Darmstadt, Germany

[#]Authors contributed equally.

*Corresponding Author: Prof. Dr. Gertrud Morlock, Justus Liebig University Giessen, Heinrich-Buff-Ring 26-32, 35392 Giessen, Germany, phone: +49-641-9939141; fax +49-641-99-39149; gertrud.morlock@uni-giessen.de

Table S1 Origin of the (a) six tin cans, (b) five thermal papers with concentration, and (c) eleven botanicals

a) Six differently coated R&D tin cans from Ceritec SRL, Metlac Group, Italy

**ID**	34	36	38	39	64	65

b) Five thermal papers collected from local retailers in Giessen, Germany, in January 2022

**ID**	**Distributor, City, Country**	***c* [g/mL]**	
A	Aldi Süd, Mühlheim an der Ruhr, Germany	0.13	
E	Esso, Echo Tankstellen, Hamburg, Germany	0.17	
O	Obi, Wermelskirchen, Germany	0.17	
R	Rewe, Köln, Germany	0.20	
V	Vision Augenoptik & Kontaktlinsen, Giessen, Germany	0.20	

c) Eleven dried botanical powders from Martin Bauer Group, Vestenbergsgreuth, Germany

**ID**	**Common name**	**Botanical name**	**Family**	**Plant part**
1	Acerola	*Malpighia glabra* L.	Malphighiaceae	fruit
2	Galangal	*Alpinia officinarum* Hance	Zingiberaceae	root
3	Hops	*Humulus lupulus* L.	Cannabaceae	blossom
4	Chamomile	*Matricaria chamomilla* L.	Asteraceae	blossom
5	Orange	*Citrus* × *aurantium* L.	Rutaceae	peel
6	Oregano	*Origanum vulgare* L.	Lamiaceae	herb
7	Rooibos	*Aspalathus linearis* (Burm. f.) R. Dahlgren	Fabaceae	leaf
8	Licorice	*Glycyrrhiza glabra* L.	Fabaceae	root
9	Thyme	*Thymus vulgaris* L.	Lamiaceae	herb
10	Hawthorn	*Crataegus* sp.	Rosaceae	leaf
11	Lemon	*Citrus* × *limon* (L.) Osbeck	Rutaceae	peel

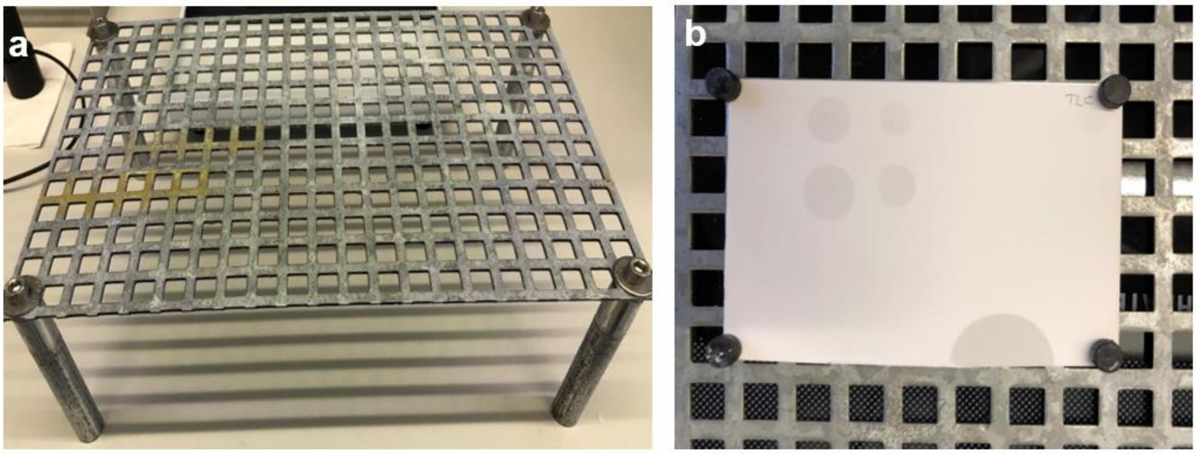

Fig. S1 Setup for plate incubation: metal perforated plate (a) and TLC plate with magnets attached to the underside of the metal plate. The construction was placed in a closed water bath, so that the distance between the TLC plate and the water was 3 cm.

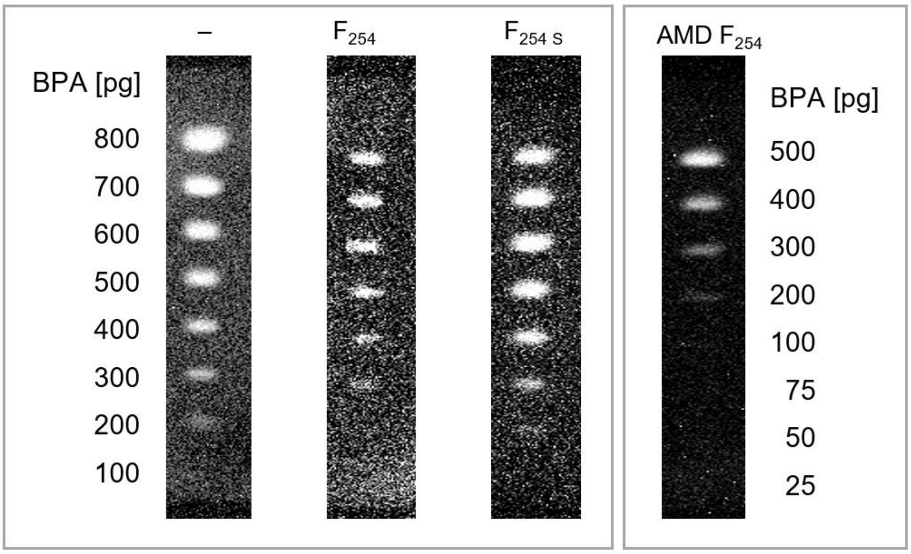

Fig. S2 Study of the bioluminescent signal response and dose-response dependency of BPA (100–800 or 25–500 pg/band) on four different types of HPTLC plates via the pA-YBS bioluminescence bioassay with a 2-h incubation; bioluminescence depicted as greyscale image with a 10-min exposure time.

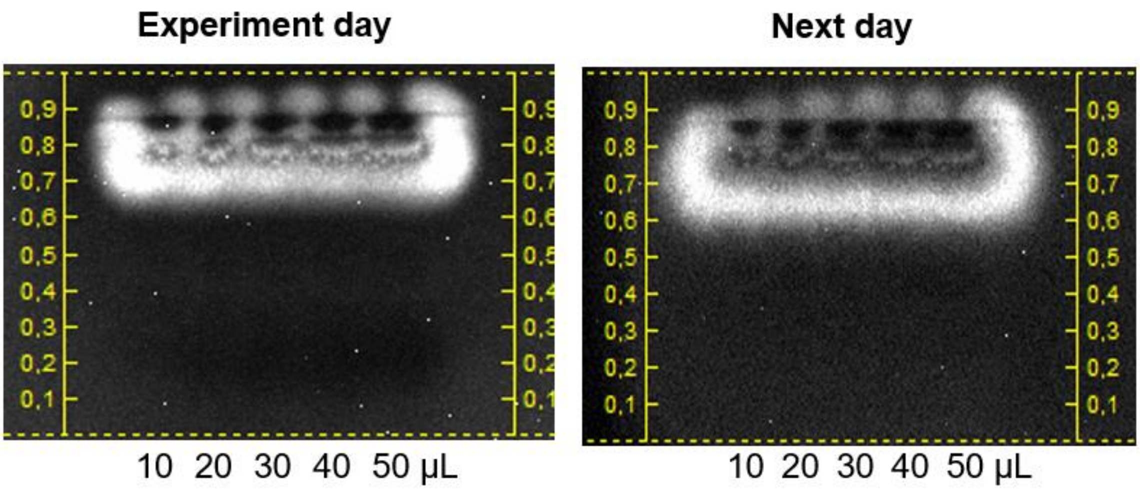

Fig. S3 Bioautogram of thermal paper V (Table 1) at different amounts (10–50 µL) on HPTLC plate silica gel 60. After the bioluminescence measurement (experiment day), the plate was dried and stored overnight. To reactivate the luciferase reaction on the next day, the plate was incubated for 20 min in the water bath (Figure S1), and then the bioluminescence was recorded with a 10-min exposure time.
